# Polymorphic and Higher-Order G-Quadruplexes as Possible Transcription Regulators: Novel Perspectives for Future Anticancer Therapeutic Applications

**DOI:** 10.3390/ph15030373

**Published:** 2022-03-19

**Authors:** Riccardo Rigo, Elisabetta Groaz, Claudia Sissi

**Affiliations:** 1 Department of Pharmaceutical and Pharmacological Sciences, University of Padova, Marzolo 5, 35131 Padova, Italy; riccardo.rigo@ceitec.muni.cz (R.R.); elisabetta.groaz@unipd.it (E.G.); 2 CEITEC—Central European Institute of Technology, Masaryk University, Kamenice 753/5, 625 00 Brno, Czech Republic; 3 KU Leuven, Rega Institute for Medical Research, Medicinal Chemistry, Herestraat 49-Box 1041, 3000 Leuven, Belgium

**Keywords:** G-quadruplex, folding landscapes, gene promoters

## Abstract

In the past two decades, significant efforts have been put into designing small molecules to target selected genomic sites where DNA conformational rearrangements control gene expression. G-rich sequences at oncogene promoters are considered good points of intervention since, under specific environmental conditions, they can fold into non-canonical tetrahelical structures known as G-quadruplexes. However, emerging evidence points to a frequent lack of correlation between small molecule targeting of G-quadruplexes at gene promoters and the expression of the associated protein, which hampers pharmaceutical applications. The wide genomic localization of G-quadruplexes along with their highly polymorphic behavior may account for this scenario, suggesting the need for more focused drug design strategies. Here, we will summarize the G4 structural features that can be considered to fulfill this goal. In particular, by comparing a telomeric sequence with the well-characterized G-rich domain of the *KIT* promoter, we will address how multiple secondary structures might cooperate to control genome architecture at a higher level. If this holds true, the link between drug–DNA complex formation and the associated cellular effects will need to be revisited.

## 1. Introduction

The long history of anticancer chemotherapy started with the identification of small molecules that were able to impair biological processes involving DNA function by either directly targeting the double helix or acting at the interface of DNA processing enzymes. These compounds showed good efficiency and many of them are still first-line treatments for several cancer diseases. However, the main drawback of these drugs lies in their low selectivity for cancer cells, with the consequent occurrence of severe off-target effects.

An entirely novel perspective for cancer therapy appeared with the discovery of telomerase, a protein soon identified as a fundamental actor in aging and cancer regulation [1]. Notably, telomerase activation has been detected in most tumor cells but not in normal cells; thus, it appears to be a suitable target for selective therapeutic approaches. In addition, it turns out that telomerase processes only on a unique DNA template, a single-stranded DNA repeat at the 3′-end of telomeres (e.g., TTAGGG in eukaryotes). In the medicinal chemistry field, these findings represented a revolution. Indeed, telomeric repeats can fold into G-quadruplexes (G4s), which are nucleic acid secondary structures that significantly differ from the canonical double helix [2]. Indeed, in G4s, canonical A-T or G-C base pairs are substituted by G-tetrads—structural elements consisting of four guanines paired through Hoogsteen hydrogen bonds that form planar arrays. These wide aromatic surfaces stack over each other, arranging in a tetrahelix with four grooves further stabilized by monovalent cations, such as K^+^ or Na^+^.

The discovery of alternative DNA folding at telomeres highlighted G4s as easily druggable targets to prevent telomerase activity, thus leading to selective anticancer therapy. Indeed, the impairment of the maintenance of telomere length was expected to be toxic only for those cancer cells that rely on telomerase to support cell survival. As a result, several drug discovery projects started to transform the already known double-stranded DNA binders into G4-targeted agents able to either selectively induce or stabilize G-quadruplex folding [3,4]. In order to direct intercalating agents towards telomeric G4s, the first strategies were devoted to increasing the ligands’ aromatic surface and decorating them with more than two cationic side chains with appropriate steric hindrance. Promising results were achieved, and several compounds with a significant preference for G4s versus the canonical double helix were identified. Consistently, these derivatives were capable of inhibiting telomerase activity while they did not largely affected other DNA processing enzymes. Nevertheless, deeper investigations of the cellular effects that occurred following drug treatment indicated a systematic unbalance of multiple biological pathways.

This general output suggested that G4s might be involved in biological processes other than telomere maintenance. Consistently, bioinformatic analyses have highlighted the fact that putative G4-forming sequences are enriched at other genomic loci besides telomeres [5,6]. More recently, the results from several ChIP-Seq, CUT&Tag, and related sequencing studies *in cells* have supported this evidence as well [7,8]. In particular, it was demonstrated that they often cluster at the proximal promoters of oncogenes. Thus, the possibility of silencing oncogene expression by inducing or stabilizing G4s outside of the telomeres was extensively explored [9,10]. This strategy presumed that the occurrence of a non-canonical structure in this region would prevent the proper recruitment of transcriptional machinery. This correlation was supported by studies in cells transfected with a plasmid where the luciferase promoter alternatively accommodated the G4-forming domain or a related mutated sequence that was unable to form a tetrahelix. The early experimentally tested examples concerned *MYC*, *VEGF*, and *BCL2* [11,12,13].

Additionally, it has been observed that specific proteins in cells can stabilize G4 formation at gene promoters. Among them, nucleolin has been identified as a G4 binding protein occurring at the *MYC* promoter and, more recently, at the promoter of the androgen receptor gene [14,15]; the overexpression of nucleolin in the cell has been confirmed to impair protein production by these genes.

It is noteworthy that, for all these models, gene silencing was amplified by cell treatments with ligands able to bind and stabilize G4s. These data provided an opportunity to drive the selective reduction in oncoprotein production by using small molecules. Unfortunately, the targeting of G4s turned out to be a multifaceted event, since several genes can accommodate G4s at their promoter; this leads to a widespread silencing of protein expression upon cell treatment with G4 binders [16]. As long as the overall reduction in protein production covers only oncogenes, the lack of selectivity might be considered beneficial for therapeutic purposes, likely resulting in a stronger anticancer efficiency and limited failure of treatment with mutations occurring at a single target site. However, this is not always the case, since G4 stabilization can silence both oncogenes and oncosuppressor genes. Moreover, although no clear rational mechanisms have been provided, it has emerged that at specific sites, G4 may even induce an increment in gene expression [16,17]. Overall, it appears that the combination of these responses might lead to unpredictable effects. This evidence further supports the need to move towards the design of targeting agents able to discriminate a single or a limited number of G4s among the large number present in living cells. As an example to summarize the complexity of this landscape in cells, it is worth mentioning that 17,950 robust G4s were recently mapped in pluripotent hESCs [18].

Since the rational design of selective targeting agents requires detailed structural information about the target of interest as well as the telomeric G4, the high-resolution structures of many G4s occurring at different genomic sites have been characterized and compared. Currently, by simply focusing on DNA G4s, 321 structures have been deposited (https://www.rcsb.org/, accessed on 24 February 2022), with the large majority being related to human sequences. Among these, the presence of two or more G-tetrads along with the occurrence of four grooves are conserved structural features. However, according to the specific primary sequences, G4s may show unique features, including, for instance, the relative arrangement of the strands, the organization of connecting loops, and the presence of capping elements. Thus, these variable tridimensional elements have become a basis for the rational design of small molecules aiming at selectively recognizing each single G4.

Despite the enormous efforts devoted to drug design that have led to the identification of a large number of G4 binders, we are still far from the goal of recognizing a single G4, and no compound is currently near to reaching clinical use.

**Figure 1:**
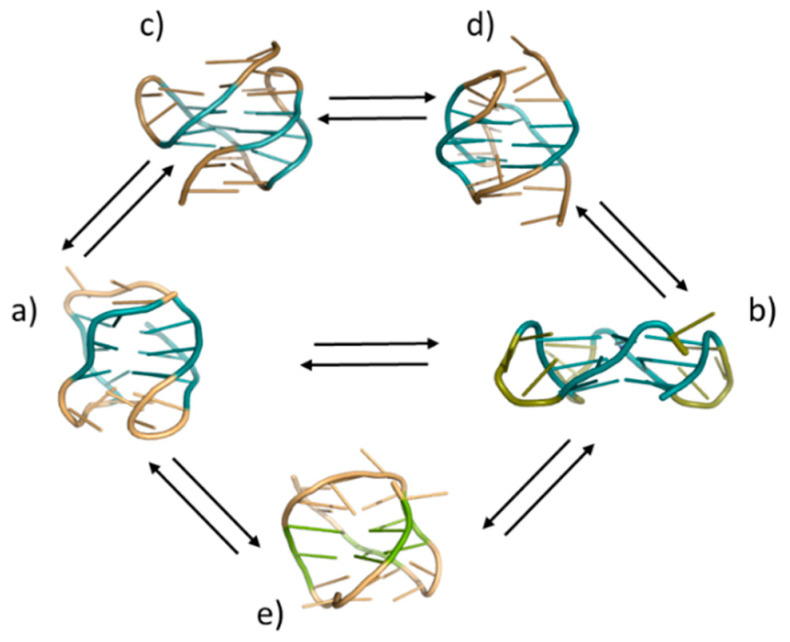
Overview of the solved G4 arrangements of telomeric repeats: (**a**) antiparallel (PDB 143D); (**b**) parallel (PDB 1KF1); (**c**) hybrid 1 (PDB 2HY9); (**d**) hybrid 2 (PDB 2JPZ); and (**e**) two-tetrads (PDB 2KKA).

A primary reason for this poor outcome lies in the polymorphic behavior of the G4-forming sequences. This feature has been known since the initial structural studies focusing on telomeric G4s were conducted. In fact, the first reported high-resolution structures of telomeric G-quadruplexes referred to a parallel and antiparallel G4 arrangement [19]. Subsequently, other telomeric G-quadruplex structures have been discovered under physiological conditions, i.e., hybrid G4s or G4s with just two G-tetrads [20,21,22,23] (Figure 1). The large number of solved structures reflects the immediate responsiveness of telomeric G4s to environmental changes (e.g., relative concentrations of metal ions, mainly K^+^ or Na^+^, and crowding conditions) and sequence frame selection, especially base composition at the 5′ and 3′ ends [24,25,26]. Even though this strategy could provide valuable tools for rational drug design, there is still a lack of accurate information on the relevance of all these conformations and their possible functions in vivo. As a result, as it was recently shown using *in cell* NMR, the recognition of G4s by small molecules in a test tube can be extensively altered in the complex intracellular environment [27].

Another issue related to the polymorphism of G4s is even more complex to unveil. Specifically, it refers to the proper evaluation of the relevance of kinetically favored species in a physiological environment.

Finally, it is noteworthy that the telomeric end consists of a single-stranded G-rich sequence ready to fold into a G4. The same does not apply to G-rich sites within the genome, where a putative G4-forming sequence is paired with a complementary strand. Thus, G4 folding at these sites requires the unwinding of the double-stranded DNA as a preliminary step. This event can be regulated and can proceed through different pathways according to the nucleic acid sequence and surrounding environment.

Here, we will summarize critical results obtained from investigations of a G-rich domain located at the proximal promoter of the *KIT* oncogene. Moreover, we will correlate these data to the acquired knowledge of telomeric sequences in order to identify the main features that make G4s valuable targets for selective anticancer therapy.

## 2. Formation of G-Quadruplex Units at the *KIT* Promoter

Several gene promoters contain putative G4-forming sequences (PQS); however, as anticipated, these sites are significantly enriched at oncogenes. Their presence at critical genomic loci can be exploited from a therapeutic point of view. However, G4 stabilization is not always sufficient to grant complete gene silencing, and, in some cases, it can even result in gene overexpression [16]. Additionally, only a limited number of deregulated oncogenes are known to drive each pathology. Thus, as is intrinsically required for any targeted approach, the accurate selection of the target is a prerequisite for setting up effective treatments.

**Figure 2:**
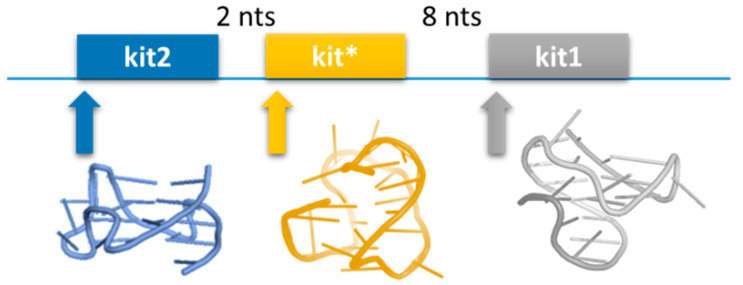
Schematic representation of the proximal promoter of *KIT* with the G4 structures that can form within this region (kit2: PDB 2KYP; kit*: PDB 6GHO; kit1: PDB 2O3M).

Among the oncogenes that contain PQS at the proximal promoter, the proto-oncogene *KIT* is highly interesting for drug design. This gene encodes a receptor kinase (c-kit) involved in controlling cell proliferation, migration, maturation, and survival. Consistently, the overexpression and mutation of this oncogene are correlated to the occurrence and sustenance of several malignancies, i.e., gastrointestinal stromal tumors, mast cell tumors, nasal T-cell lymphomas, seminoma/dysgerminoma, and acute myeloid leukemia [28]. The proximal promoter of *KIT* contains at least three G-rich sequences whose potential to fold into G4s and control gene expression has been confirmed (Figure 2). It is noteworthy that the high-resolution structures of the G4 adopted by these sequences have been obtained and shown to exhibit specific structural features making each of these units a unique structural domain. Both kit1 and kit2 fold into a parallel G4, but they are still very different from each other. Within the G4 structure assumed by kit1, one spare guanine is recruited to complete a G-run, forming a wide cleft [29]. Conversely, two different species, a monomeric and a dimeric structure, have been reported for kit2 G4 [30,31]. Finally, kit* folds into a monomeric two-tetrad antiparallel G4 that is further stabilized by capping elements, in particular, a stable GC Watson–Crick base pair stacking below the 5′-tetrad [32].

Finally, kit1, kit2, and kit* are closely located, with only a few nucleotides separating each other.

These structural data prompted multiple virtual and in vitro screenings of small molecules to identify new entities with the ability to stabilize the G4 arrangements occurring at the *KIT* promoter. A direct correlation between G4 induction/stabilization and a reduction in the overexpression of c-kit has been systematically observed [33,34,35,36]. However, no compounds have appeared to be highly selective for *KIT*-related G4s compared to those occurring at other sites, nor do they discriminate among the solved G4 of *KIT* promoter [37,38].

Unveiling the G4 structures among those resolved that can fold in the intracellular environment is an essential requirement to determine the functional roles of G4s in biological processes and, eventually, guide our drug design efforts.

## 3. Differential Folding Landscapes of G-Quadruplexes at the *KIT* Promoter

As anticipated, the dissection of the G-quadruplex folding landscape is a relevant piece of information when mapping the structural evolution of PQS in the nuclear environment. Indeed, folding intermediates, which are structurally distinct from the characterized thermodynamically favored species, could show half-life times compatible with the timescale of physiological processes [39]. Considering this aspect, we cannot rule out that the control of biological functions in the cell might be driven by the kinetically favored G4s, rather than the more thermodynamically stable G4s. The main difficulties in addressing this point lie in properly identifying the number of folded/misfolded species, characterizing their structures, and quantifying their relative distribution over time. A relatively large amount of data has been collected using different models, with the telomeric sequence being most commonly represented [40,41,42]. These studies confirmed the occurrence of other transient species besides those already solved, increasing the number of known telomeric DNA conformations (see Figure 1).

**Figure 3:**
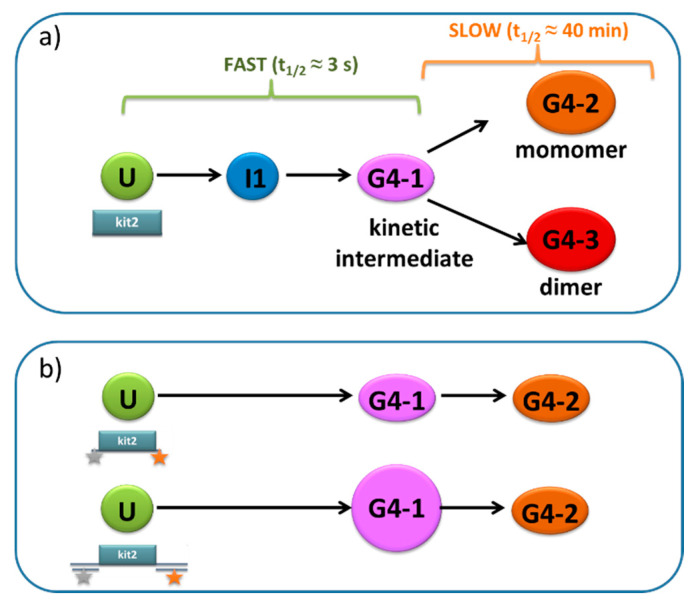
Folding of kit2 as determined in bulk (**a**) or by single molecule FRET (**b**) analyses. U refers to the unfolded (or pre-folded) state; I2 to an uncharacterized short-lived intermediate; and G1, G2, and G3 to a kinetically favored, a monomeric, and a dimeric G-quadruplex, respectively.

When considering the sequences located at gene promoters, it should be kept in mind that, as observed for *KIT*, multiple PQS domains might be present that commonly are not conserved in terms of their primary sequence and, consequently, folding landscape. In solution studies focusing on the studied sequences located at the promoter of *KIT* indicated that the folding of kit* and kit1 into a G4 is fast, and the final species correspond to the solved structures. Conversely, kit2 shows a complex folding pathway. First, the wild-type sequence folds into a monomeric and dimeric G4, whose relative abundances in solution are correlated to the oligonucleotide concentration. Moreover, the folding process can be divided into a fast-folding step and a subsequent slow-folding step (Figure 3a). This pathway is similar to that reported for the telomeric sequence [43]. The first step is completed within seconds and involves at least one G-quadruplex form (G4-1 in Figure 3a), while the second folding step leads to the formation of the above-mentioned monomeric and dimeric parallel G4s (G4-2 and G4-3 in Figure 3a, respectively). The final species do not interconvert one into the other [44]. Only monomeric species are expected to be relevant in physiological conditions. However, the high efficiency of dimer formation prevents the acquisition of unbiased evidence regarding the folding process of the monomeric forms only.

Properly designed single-molecule analyses can help to address this issue. In a recent study where FRET analyses were performed, a low sample concentration in solution prevented the occurrence of dimer formation [45]. As reported in Figure 3b, two different constructs were designed. In the first construct, the fluorescent labeling groups were attached at the 5’- and 3′-end of the G-rich domain, mimicking a model previously used for kinetic analyses in bulk. In the second construct, the FRET pair was inserted within two double-stranded flanking domains added at the terminals of the G-rich core. This model was designed to represent the formation of the G4 within the double-stranded promoter region. As expected, in the absence of K+, both constructs behaved as an unfolded system. Conversely, in the presence of the cation, three main species were identified: one corresponding to a residual fraction of unfolded DNA (U), and two characterized by higher FRET efficiencies, conceivably related to the folded species. Following their kinetic formation allowed for their association with the two monomeric G4s (the fast- and slow-forming G4-1 and G4-2, respectively) previously characterized through bulk experiments. It is noteworthy that these FRET analyses highlighted an increment in the relative concentration of G4-1 vs. G4-2 in the longer construct, further supporting the relevance of the kinetic intermediate in the physiological environment. A setup of magnetic bead constructs designed to follow the G4 folding of kit2 embedded in a transient DNA duplex further supported the relevance of the surrounding dsDNA environment in modulating the kinetics and stability of G4s [46]. This correlation can be extended to any G-rich sequence, although the interplay of the two different structural domains is, again, largely modulated by the selected G4-forming sequence.

## 4. Conformational Selection of G-Quadruplexes as a Tool for Selective Functional Targeting

The folding landscape described above further supports the occurrence of two different physiologically relevant monomeric G4 arrangements at kit2. It can be assumed that both might represent suitable targets for silencing oncogene expression. Still, the results described above do not provide any clues concerning which arrangement might be more useful as a target for a drug design program.

This issue is a well-known bottleneck in medicinal chemistry. To dissect this point, a useful approach consists of using small molecules to promote a conformational selection and trap a single G4 conformation; from this perspective, some ligands that are able to target a unique state of the telomeric G4 have been tested [47,48].

**Figure 4:**
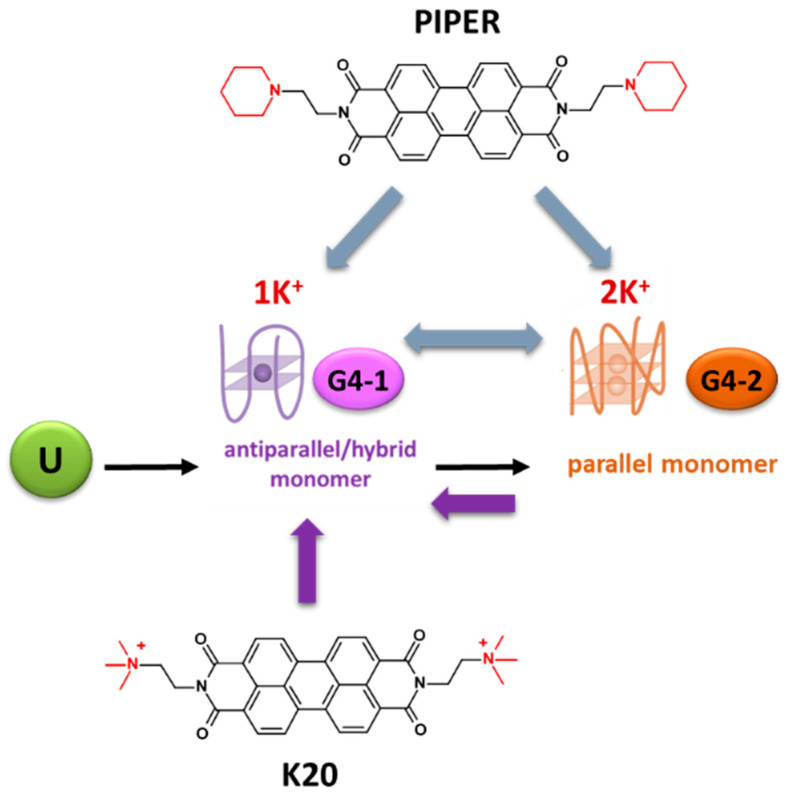
The two G4 states of kit2. 1K^+^ and 2K^+^ refer to the number of K^+^ ions bound to the folded G4. U refers to the unfolded (or pre-folded) state, and G1 and G2 to the kinetically favored and the structurally solved monomeric G-quadruplex, respectively.

This approach, which could also be applied to target kit2, was tested by selecting two different perylene derivatives, namely PIPER and K20 [49]. Previous studies have highlighted that these two structurally related compounds do not form similar complexes with the telomeric sequence [50]. When tested in the presence of kit2, a native mass spectrometry analysis showed that G4-1 and G4-2 were the most abundant folded species in solution at a low potassium chloride concentration. When PIPER, a well-known G4 binder, was included in the reaction, both species were recognized and their relative distribution was conserved. Conversely, in the presence of its derivative K20, the equilibrium was shifted mainly towards the stabilization of G4-1 (Figure 4). Freezing this intermediate allowed us to better understand its structural features. In this case, the native mass data and chiroptical signature address G4-1 as a two-tetrad G4, which is compatible with an antiparallel arrangement.

These results offer a new perspective to define the relevance of different G4 topologies within a physiological environment. Indeed, this approach can provide new tools to selectively dissect the functional consequences of stabilizing specific folding intermediates in cells. Unfortunately, PIPER and K20 are not selective ligands for kit2. Indeed, as mentioned above, they can efficiently bind other G4 sites. In addition, they retain a relevant affinity for dsDNA. As a result, it can be expected that their use in the cell would not provide an output strictly related to the discussed specific conformational selection. Moreover, as suggested by the herein tested derivatives, the differential stabilization of one or more G4s depends on a very limited selection of structural features, thus further highlighting the difficulty of systematically predicting the preferences for a single G4 form within a polymorphic mixture.

## 5. Hierarchical Organization of G4 Repeats

The repetitiveness of G-rich sequences represents a peculiar feature of telomeres. As a consequence, the single-stranded 3′-end of telomeres can accommodate multiple G4s. In this case, they can fold as independent units (beads-on-the-string model) or mutually interact (cross-talking beads model). Independent from the preferred folding model of the telomeric sequence, a further issue is associated with the tendency of these long repeats to form the maximal number of G4 units or not. Currently, accumulating evidence points to the formation of the maximal number of G4 units: this corresponds to the occurrence of several folded G4s spatially close to each other [51,52].

The final telomeric assembly can be considered as a higher-order G4 tridimensional architecture that can be exploited for selective targeting [53,54].

The occurrence of repetitive G-rich sequences and the consequent formation of repetitive G4s is a property shared by a limited number of G-rich regions within the genome. A significant example is the insulin-linked polymorphic region (ILPR), located upstream the transcriptional start site of the insulin gene, where the most prevalent tandem repeats correspond to the (ACAGGGGTGTGGGG) sequence [55].

It is also possible to identify a small subset of gene promoters where many G4s presenting different sequence compositions and topology are closely clustered. An intriguing example is found in the hTERT promoter, where the formation of three contiguous G4s can compete with an arrangement where two G4s are separated by one hairpin [56,57].

**Figure 5:**
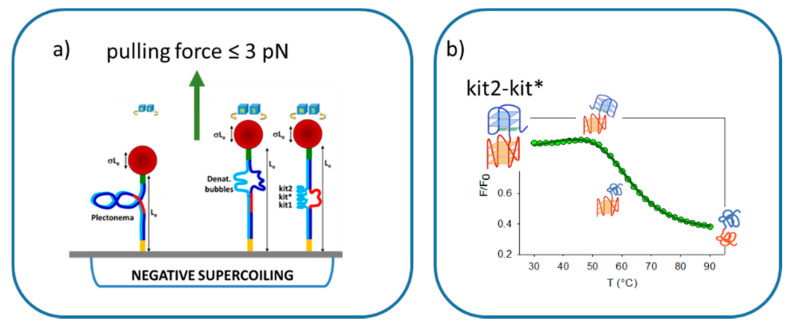
Multiple G4s at the *KIT* promoter: (**a**) model of the magnetic tweezer approach used to monitor G4 formation (adapted from [59]) and (**b**) proposed unfolding pathway of the kit2-kit* fragment (adapted from [60]).

A critical feature differentiating the G-rich sequences at the telomeres and promoters is the localization of the latter within a double-stranded DNA template. Multiple experimental techniques have confirmed that G4 formation is not limited to the telomeric sites in living cells [58]. However, the ability of promoters to accommodate the maximal number of G4s in physiological conditions is still under investigation. Since the G-rich domain of the *KIT* promoter fits into this frame, it represents an excellent model to address this issue. Thus, the G-rich domains of the *KIT* promoter were inserted within a long dsDNA frame to be studied at the single-molecule level by a magnetic tweezer approach (Figure 5a). As a novelty, a low pulling forces regime was selected [59]. Under these experimental conditions, the relaxed double helix could not unwind. However, under supercoiling conditions, the applied forces were sufficient to convert the so-formed supercoiled DNA into a denaturation bubble. The rationale for this design lies in the fact that the formation of G4s can occur after duplex unwinding, since the low applied forces cannot unfold them. Based on the energy applied to unwind the template, the size of the denaturation bubble, and their dependence upon the presence of K^+^ in the sample, it was possible to support the simultaneous formation of three G4s at the G-rich domain of the *KIT* promoter.

As anticipated, the formation of multiple G4s can lead to a direct interaction between the folded domains. This interaction may influence both the folding topology and overall stability of the tetrahelical arrangements, thus representing a further attractive site for intervention by small molecules. As far as the telomeric repeats are concerned, the most recent evidence points to a full range of possible organizations of the repeating G4 units. The occurrence of a G4:G4 interface is transiently present [52]. The same was foreseen for the kit2-kit* sequence due to the short G4–G4 connecting loop (three nucleotides) corresponding to the spacing of the telomeric G4 units. Although the topology of kit2 and kit* were significantly different with respect to that of the telomeric sequence, a spectrophotometric investigation of this sequence supported an interaction between the two G4 domains of *KIT* (Figure 5b) [60]. Notably, this might involve one cupping element of the kit* domain that, in this way, can use the kit2 external tetrad as a sort of template surface [61].

This emerging picture is consistent with the existence of a G4–G4 interaction. However, the low interaction energy indicates that G4–G4 crosstalk is a transient structural element that will be difficult to properly exploit for an efficient rational drug targeting approach; however, it is still worthy of further investigation [62].

## 6. G4 Repeats and Protein Recruitment *in Cell*

The potential targeting of G4s is highly attractive since the conversion of nucleic acids into a such structure represents a signal that can regulate protein recruitment at defined genomic sites [63,64]. Remarkably, some proteins either recognize and process G4s or are displaced from the nucleic acid upon G4 formation; others, such as Yin and Yang 1, may use spatially distant G4 units to drive long-range DNA looping [65]. These variegate models suggest that we should consider G4s as “epigenetic” regulatory elements. Several examples have been reported in terms of the recruitment/displacement of transcription factors at specific loci. As an example, considering the kit2kit* G-rich sequence, it was confirmed that the affinity ranking for the transcription factor SP1 was kit2 < kit* < kit2kit* [66,67]. However, this ranking order referred to the binding of the transcription factor to double-stranded sequences. In the presence of G4 substrates, the residual DNA–protein interaction was very weak in all cases, thus hampering the possibility of quantifying the contribution of the tandem G4 formation to SP1 binding.

**Figure 6:**
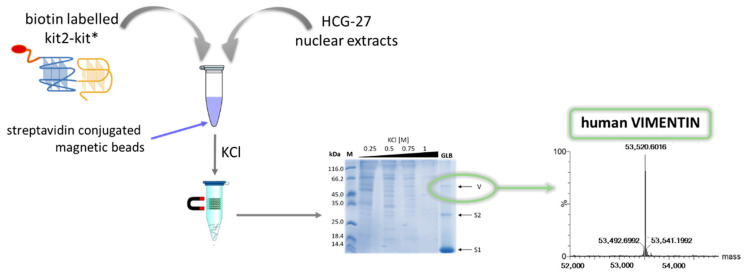
Outline of a pull-down approach leading to the discovery of vimentin as a G4 repeat selective binder (adapted from [68]).

Nevertheless, due to the above-mentioned variable features and the stability of G4 repeats in contrast with isolated G4s, it is intriguing to consider that these two groups of secondary structures may play distinct roles in the cells. However, no data are available concerning any relevance of G4 repeats as functional entities that are actively involved in regulatory functions (i.e., telomere capping and gene transcription and translation). Studying the proteins that are able to selectively recognize G4 repeats could be a strategy to address the specific functions of these DNA structures. Pull-down experiments on nuclear extracts were performed to identify proteins that preferentially bind G4 repeats rather than isolated G4s (Figure 6) [68]. Remarkably, it turns out that vimentin, an intermediate filament protein, can bind G4 repeats with high affinity. In contrast, it does not recognize isolated G4s nor the same G-rich sequences when arranged in single- or double-stranded forms. An investigation of the relevance of this interaction is still underway. Preliminary data indicate a significant correlation between the proteins expressed by genes containing G4 repeats at the proximal promoter and the established biological activity of vimentin. This evidence opens a completely new range of possibilities. Since the higher-order architectural organization of G4s selectively recruits proteins, it is possible to exploit the protein–DNA interface as a novel target site. In this scenario, it is important to underline that vimentin is one of the most relevant factors supporting the epithelial–mesenchymal transition and providing mobility to cancer cells; it is consistently considered as an indicator of poor prognosis. Thus, the remodeling of its DNA binding profile in cancer cells may lead to the design of novel therapies that are able to block the metastatic behavior of many cancers.

## 7. Conclusions

Overall, the findings summarized here show how the scope of targeting G4s for therapeutic applications has evolved with time. The original goal has clearly changed, as the initial design devoted to recognizing G4s over double-stranded DNA was replaced by the necessity to distinguish a single G4 (or a subset of functionally related G4s) from a large number of G4s. This turned out to be an even more ambitious goal due to the limited degree of variability that is frequently not relevant in the dynamic physiological environment. However, the more we expand our knowledge of the structural equilibria governing these fascinating nucleic acid domains, the more precise our view of potential targetable features will be. A highly attractive feature is the higher-order G-quadruplex organization. As revealed by our data on vimentin, nature has most likely already given good clues to help us along this pathway. As mentioned earlier, a closer look at the complex biomolecular networks involving G4s could provide important insights that lead to new opportunities for safer and more efficient treatments for oncological patients. Furthermore, it is worth mentioning that this approach could be extended to other therapeutic fields. Indeed, it is important to note that comparable conditions occur at specific coding regions, i.e., the hexanucleotide repeats (GGGGCC)n in the first intron of the C9orf72 gene. Here, the expansion of the repeats is the most frequent cause of both familiar and sporadic amyotrophic lateral sclerosis (ALS) [69]. Thus, the investigation workflow presented here for the telomeric sequence and promoter of *KIT* should be extended to other genomic domains as well as to the RNA landscape.

## Data Availability

Data sharing not applicable.
